# Morphological characteristics of lenticulostriate arteries in a large age-span population: Results from 7T TOF-MRA

**DOI:** 10.3389/fneur.2022.944863

**Published:** 2022-07-22

**Authors:** Ning Wei, Jing Jing, Yan Zhuo, Zihao Zhang

**Affiliations:** ^1^China National Clinical Research Center for Neurological Diseases, Beijing Tiantan Hospital, Capital Medical University, Beijing, China; ^2^Tiantan Neuroimaging Center of Excellence, Beijing Tiantan Hospital, Capital Medical University, Beijing, China; ^3^Department of Neurology, Beijing Tiantan Hospital, Capital Medical University, Beijing, China; ^4^State Key Laboratory of Brain and Cognitive Science, Institute of Biophysics, Chinese Academy of Sciences, Beijing, China; ^5^Institute of Artificial Intelligence, Hefei Comprehensive National Science Center, Hefei, China; ^6^University of Chinese Academy of Sciences, Beijing, China

**Keywords:** lenticulostriate artery, TOF-MRA, 7 Tesla, aging, subcortical nuclei

## Abstract

Lenticulostriate arteries (LSAs) originate from the middle cerebral artery (MCA) and supply blood to the basal ganglia. The evaluation of its structure and function is essential for the etiological diagnosis of subcortical infarction. However, the characteristics of LSA in a healthy population remain poorly described. Our study aims to acquire morphological measurements of LSA by 7T TOF-MRA on 140 healthy volunteers with a large age range (21–68 years). The results show that the number of branches of LSA gradually decreases with age (*r* = −0.328, *p* < 0.001), and the maximum visible length becomes shorter (*r* = −0.385, *p* < 0.001). Moreover, there is a significant correlation between the volume of the basal ganglia nuclei and the morphology of LSA. The volume of the putamen is associated with the number of stems (*r* = 0.267, *p* < 0.001) and branches (*r* = 0.236, *p* < 0.001) of LSAs, while the volume of caudate is closely related to the maximum visible length of LSAs (*r* = 0.199, *p* = 0.001). In conclusion, this study is the first *in-vivo* study to report the morphology of LSA with a large sample size of more than one hundred cases. These findings are valuable in understanding the degeneration of LSAs during aging.

## Introduction

Lenticulostriate arteries (LSAs) arise from the middle cerebral artery (MCA). They are small perforating arteries that supply blood to important subcortical areas, including part of the basal ganglia and posterior limb of the internal capsule (1–3). The occlusion or stenosis of LSA leads to small infarcts or lacunar stroke, which should be distinguished from large-artery atherosclerosis in the etiologic diagnosis of stroke (4, 5). Therefore, assessing the structure and function of LSAs has important clinical implications.

Traditionally, digital subtraction angiography (DSA) is considered the “gold standard” in vascular imaging. However, it is not the ideal methodology for imaging LSA because of its invasiveness. Magnetic resonance imaging (MRI) can provide non-invasive cerebral angiography and facilitate the detection of vascular abnormalities. However, time-of-flight MR angiography (TOF-MRA) in clinical settings fails to display cerebral small vessels like LSA because of the limited signal-to-noise ratio (SNR) and flow-related enhancement. The development of magnetic field brings in a higher SNR and increased tissue contrast at ultra-high field (≥7 Tesla, 7T). Benefiting from stronger flow-related enhancement, TOF-MRA at 7T achieves higher spatial resolution that is capable of imaging perforating arteries, allowing the researchers to investigate LSA non-invasively (6–9). The abnormalities of LSAs have been observed in ischemic stroke, hypertension, and hereditary cerebral small vessel disease (10–13). These findings suggest that 7T TOF-MRA can be the first-line imaging modality for LSA, especially when DSA is infeasible, such as in patients allergic to contrast media and healthy volunteers.

While TOF-MRA at 7T is gradually being accepted by clinicians as an imaging method for LSA, the characteristics of LSA in a healthy population remain poorly described. Before the radiological method was developed, the morphological measurements of small arteries like LSAs could only be obtained by autopsy (3). DSA provided knowledge of the whole-brain vascular network but is usually collected only in patients with cerebrovascular diseases. Therefore, previous studies mainly focused on patients with neurovascular disease and healthy elderly people (14). The morphological measurements of LSAs in different age groups are still lacking. On the other hand, low concordance rates in terms of vascularized territories were found among autopsy studies investigating the LSAs (15). Large variability was demonstrated in the configuration of cerebral perforating arteries. As a result, the lack of morphological norms hinders the application of 7T TOF-MRA in clinical settings to identify LSA abnormalities.

In this study, we aim to collect images of LSAs by 7T TOF-MRA on healthy volunteers with a large age range and measure its morphological characteristics, including the counts, lengths, and distributions. The overviews of these metrics and their changes with age will be investigated. The spatial relationship between LSAs and basal ganglia nuclei will be explored.

## Materials and methods

### Participants

This study recruited one hundred and forty healthy volunteers (64 males). The participants were screened to form a normal distribution of their ages (<30 years: 13, 30–39 years: 40, 40–49 years: 33, 50–59 years: 38, >60 years: 16). Informed consent was obtained from all participants and was approved by the local institutional review board. All the subjects received a clinical diagnosis or medical consultation to exclude cerebrovascular disease within one month before participating in the experiment. The 7T MR images of the subjects were evaluated by two neurologists with three years of experience to further rule out cerebrovascular or other clinically relevant abnormalities.

### Magnetic resonance imaging

All MRI scans were performed on a 7T whole-body research MR scanner (Siemens Healthcare, Erlangen, Germany). A birdcage transmission / 32-channel receiving head coil (Nova Medical, MA, USA) was used for all scanning procedures.

T1-weighted magnetization-prepared rapid gradient echo (T1w-MPRAGE) and TOF-MRA were collected for every participant. The T1w-MPRAGE was obtained for structural images, with the following parameters: FOV = 224 × 224 × 179 mm^3^, resolution = 0.70 × 0.70 × 0.70 mm^3^, TR = 3000 ms, TE = 3.23 ms, inversion time = 1050 ms, FA = 8°, BW = 320 Hz/Px, acceleration factor (generalized auto-calibrating partial parallel acquisition, GRAPPA) = 3, acquisition time (TA) = 5 min 54 sec. The TOF-MRA sequence was optimized to visualize LSAs. The imaging slab was positioned obliquely axial covering the lower bound of the MCA and the upper bound of the basal ganglia. The imaging parameters were FOV = 180 × 135 × 52 mm^3^, resolution = 0.23 × 0.23 × 0.36 mm^3^, TR = 15 ms, TE = 3.57 ms, FA = 20°, BW = 151 Hz/Px, GRAPPA factor = 2, TA = 8 min 20 sec. During the experiment, all the images were visually checked by the data collection group, including one neurologist and two experienced MRI operators. One repeated scan was required when motion corruption or other artifacts were identified by two or more people in the group.

### Image analysis

All images were post-processed to get the morphological measurements of LSAs and subcortical nuclei. The distribution of LSAs in subcortical nuclei was investigated using statistical analysis.

#### Morphological characteristics

**Figure 1:**
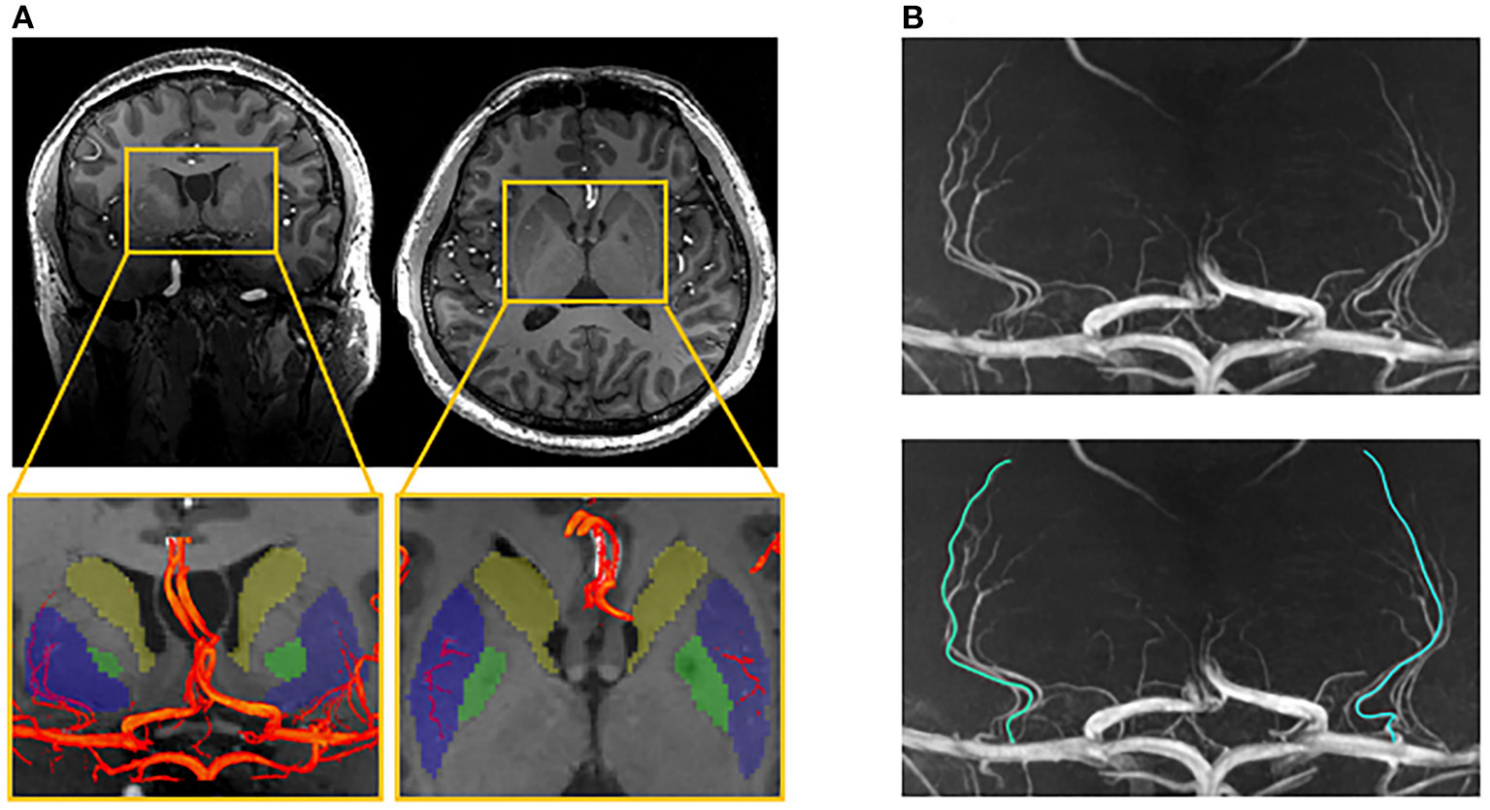
Illustration of the measurements of lenticulostriate arteries (LSAs). **(A)** The LSAs (red) and their supplying territories (basal ganglia), overlaying on the structural image (blue: putamen; green: globus pallidum; yellow: caudate); **(B)** The coronal maximal intensity projection of TOF-MRA, on which the curved length of LSA was measured.

Three-dimensional (3D) MRA images were reviewed and analyzed using certificated medical imaging software (Osirix MD, Pixmeo SARL). For the optimal representation of LSAs, the images were reconstructed by maximum intensity projection (MIP) in the coronal view with a slab thickness of 25 mm. On the reformatted coronal MIP and raw images, the number of LSA stems and branches was counted by an experienced neurologist with three years of experience. Stems were defined as LSAs directly connected to the first segments of anterior cerebral arteries (A1) or middle cerebral arteries (M1). Branches were defined as daughter vessels arising from parent LSA stems without any subordinate branches. If a trunk had no branches, it was regarded as both stem and branch. The maximal length was measured on the longest LSA in the coronal MIP view, which was the curved length from MCA to the visible end, as shown in Figure 1. It was worth noting that the measurement was the projected length of LSA in the coronal MIP view. Nonetheless, the projected length reflected the length of LSA in 3D space. The number of stems and branches and the maximal length were obtained in both hemispheres of each subject.

#### Subcortical regional volumes

We used Freesurfer 7.0 to estimate the volumes of basal ganglia (BG), including putamen, caudate, and globus pallidum. Since the images of 7T MRI were highly affected by the spatial variation of the magnetic field, the automated segmentation by the conventional method for T1w-MPRAGE underperformed. Recently, a new segmentation method called SynthSeg was reported (16). It used a convolutional neural network to segment structural images of various contrast and resolutions without retraining or fine-tuning. This method successfully overcame the segmentation fault caused by field inhomogeneity on 7T T1w-MPRAGE and had a stable and accurate performance. Therefore, we used SynthSeg on our T1w-MPARAGE images for the segmentation of subcortical nuclei. The results of all subjects were manually checked to avoid segmentation failure. These subcortical structure volumes were further compared and analyzed with the morphological characteristics of LSAs.

### Statistical analyses

For each subject, age, gender, the morphologic characteristics of LSA, and the volumes of three subcortical structures on each hemisphere were analyzed. All the quantitative data were presented as means ± standard deviations (SDs). First, the paired t-test was used to compare the characteristics of LSAs in the left and right hemispheres. In the subsequent analysis, the numbers of stems and branches of LSA in both hemispheres were added up, and the maximum visible length of LSA was averaged to represent the LSAs of the whole brain. The gender difference in the morphology of LSA was also compared. Using Pearson's correlation coefficient, the age-specific trends of the branches, stems, and maximum visible length of LSA were revealed. The linear regression analysis was used to predict the changes in LSA characteristics with age. Adjusted for age, gender, and brain volume, partial correlation analysis was used to examine the relationship between morphological measurements of LSA and volumes of subcortical nuclei. All calculations were performed using SPSS vision 21 software with an α level of significance of 0.05. Bonferroni correction was applied to the statistical results to reduce type-1 errors caused by multiple comparisons.

## Results

A total of 137 image volumes passed the quality control and entered the post-processing pipeline (mean±SD: 44 ± 11 years; range: 21–68 years; and female/male: 75/62). The means and standard deviations of morphological measures of LSA in both hemispheres are shown in Table 1. Paired samples *t*-tests were conducted to evaluate the lateralization of LSA structures. The results showed no significant difference in the branches, stems, and maximum visible length of LSA between the left and right hemispheres, indicating the non-lateralized distribution of LSA.

**Table 1:** The characteristics of LSA in the left and right hemispheres.

	**Left** **(mean**±**SD)**	**Right** **(mean**±**SD)**	***p*** **-value**
Stem (n)	2.64 ± 0.92	2.56 ± 0.82	*t*(136) = 0.88, *p* = 0.38
Branch (n)	4.77 ± 1.60	4.61 ± 1.36	*t*(136) = 1.52, *p* = 0.13
Length (cm)	3.36 ± 0.60	3.38 ± 0.55	*t*(136)= −0.47, *p* = 0.64

The characteristics of LSA were compared between men and women, as shown in Table 2. The independent sample *t*-test showed no gender difference in the number and length of LSA.

**Table 2:** The characteristics of LSA between men and women.

	**Male** **(*****n*** = **62)**	**Female** **(*****n*** = **75)**	***p*** **-value**
Stem (*n*)	5.07 ± 1.38	5.31 ± 1.49	*t*(135) = −0.98, *p* = 0.33
Branch (*n*)	9.18 ± 2.53	9.55 ± 2.80	*t*(135) =-0.80, *p* = 0.42
Length (cm)	3.42 ± 0.50	3.33 ± 0.50	*t*(135) = 1.02, *p* = 0.31

### Age-related changes in morphological measurements

**Figure 2:**
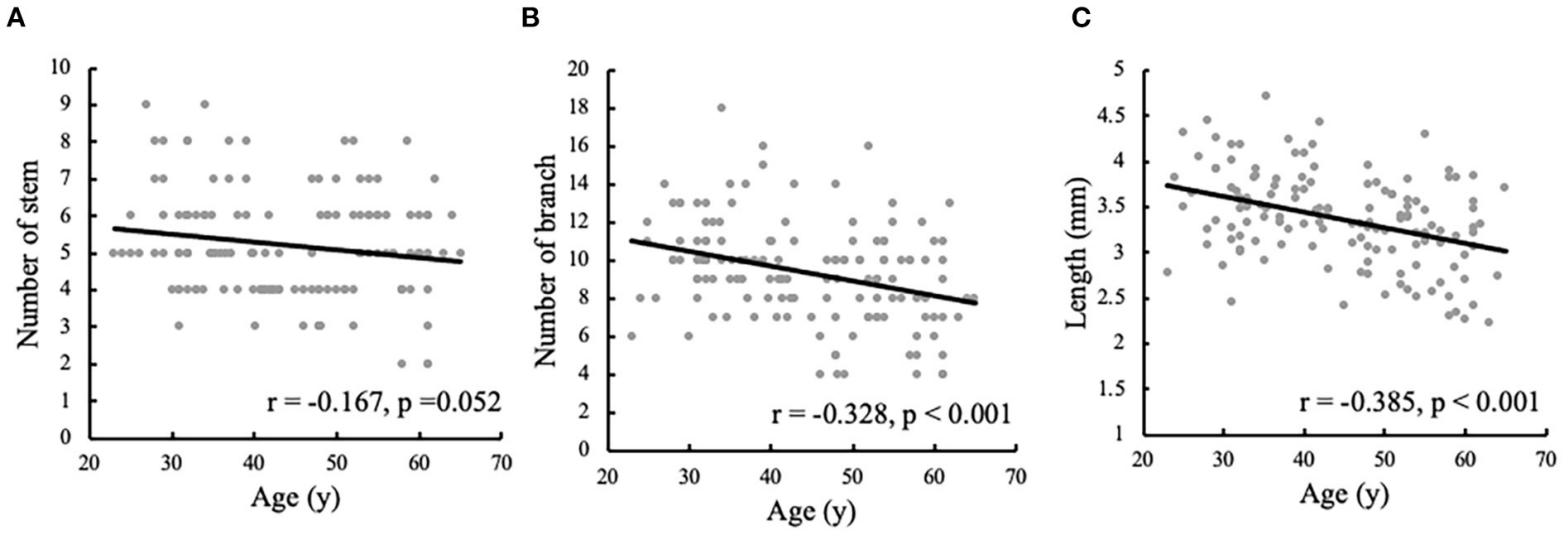
Correlation analyses of the morphological measurements of lenticulostriate artery (LSA) and the ages. The correlations are shown between the ages and **(A)** the number of LSA stems, **(B)** the number of LSA branches, and **(C)** the maximum visible length of LSA.

The associations between age and the morphological measurements of LSA are shown in Figure 2. During the aging process, both the number of branches (*r* = −0.328, *p* < 0.001) and maximum visible length (*r* =-0.385, *p* < 0.001) of LSA significantly decreased. Meanwhile, a weak association was found between the reduced number of stems and the age (*r* = −0.167, *p* = 0.052). When evaluating the effect of age on the change rate of branch count and maximum visible length (branch-age interaction and length-age interaction) by linear regression analysis, we found that the branches of LSA decreased by 0.078 (95% CI: −0.117, −0.04; *p* < 0.001) and the length of LSA decreased by 0.017 cm (95% CI: −0.024, −0.010; *p* < 0.001) per year of age. To better understand the characteristics of LSA in different age ranges, the number of stems, branches, and the maximum visible length of LSA were further divided into different age groups, which were summarized in Table 3. The decreasing trends of branches and maximum visual length with age can be easily observed.

**Table 3:** The characteristics of LSA in different age groups.

**Age (years)**	**20**–**30**	**30**–**40**	**40**–**50**	**50**–**60**	**60**–**70**
Number (*n*)	13	40	32	38	14
stem (*n*)	6.23 ± 1.42	5.53 ± 1.39	4.38 ± 1.13	5.34 ± 1.36	4.79 ± 1.53
branch (*n*)	10.69 ± 2.36	10.50 ± 2.54	8.59 ± 2.59	8.97 ± 2.44	7.86 ± 2.71
length (cm)	3.72 ± 0.50	3.53 ± 0.44	3.38 ± 0.44	3.18 ± 0.49	3.05 ± 0.52

### Basal ganglia volumes in the vascular territories

**Figure 3:**
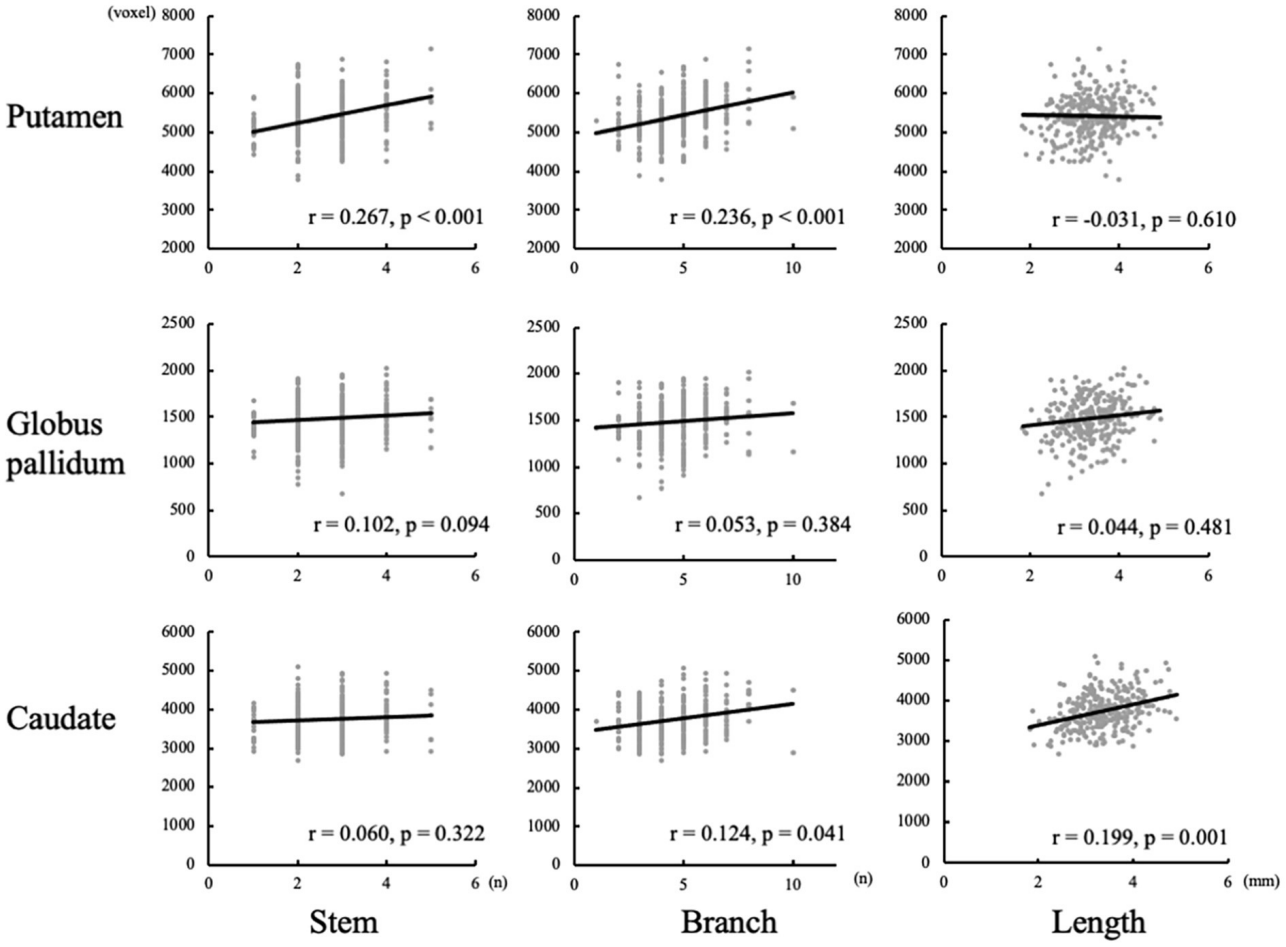
Correlation between basal ganglia volumes and measurements of lenticulostriate arteries (LSAs).

After controlling for age, gender, and ipsilateral hemisphere volume, the relationship between LSA morphology and subcortical nuclei was assessed, as shown in Figure 3. The volume of putamen was found to be positively correlated with the number of stems *(r* = 0.267, *p* < 0.001) and branches (*r* = 0.236, *p* < 0.001) of LSA. There was a positive correlation between caudate volume and the maximum visible length of LSA (*r* = 0.199, *p* = 0.001). But no significant association was found between globus pallidum and LSA.

## Discussion

In this study, we used 7T TOF-MRA for the non-invasive depiction of morphological characteristics of LSA in a healthy population. To our knowledge, this is the first *in-vivo* study to report LSA morphology with a large sample size of more than one hundred cases. The results demonstrate the absence of hemispheric lateralization or gender difference in LSA distribution. In adults, the number of stems and branches of LSA gradually decreases with age, and the maximum visible length becomes shorter.

Our findings are consistent with some previous studies in which the number of perforators decreased over the age of 60 years (8). A phase-contrast MRI study found decreased flow velocity of LSA in the elderly, which appears to reflect the arteriosclerotic changes of the aging cerebrovascular system (13, 17). The lower flow velocity explains the decreasing trends of the count and visible length of LSA, as the contrast of TOF-MRA comes from the flow-related enhancement by the moving spins of the blood. Notably, in previous clinical studies, significant decreases in the number of LSAs were reported in various diseases, such as hypertension, cerebral small vessel disease, and subcortical vascular dementia (10–12). This study suggests that the morphological changes of LSA due to normal aging must be considered in the study design of related diseases. Strict age-matching criteria should be used when recruiting the control group. In addition, the morphological features of LSA caused by pathology and normal aging need to be further distinguished to improve the accuracy of the radiological diagnosis of related diseases.

This study also reveals the relationship between the morphology of LSA and the volume of BG, which is the supplied territory of LSA. We found that the volume of putamen was associated with the number of LSAs, while the volume of caudate was closely related to the length of LSAs. These findings are reasonable considering the anatomy that most of the LSAs go through the putamen and caudate. As caudate locates at a higher axial position than other nuclei of BG, only the LSAs with rich blood flow have sufficient contrast to be displayed by TOF-MRA, resulting in the correlation of visible length with caudate volume. Based on their origins, LSAs can be divided into medial and lateral groups. Most of the perforators visualized by non-contrast 7T TOF-MRA are lateral LSAs, while post-contrast TOF-MRA has greater performance in displaying medial LSAs (18). The putamen is mainly supplied by lateral LSAs and its volume shows a correlation with the vascular counts on TOF-MRA, while globus pallidum supplied by medial LSA exhibits no correlation with the number of LSAs.

Both the volume of BG and the number and length of LSA are found to decrease with age. It reflects that LSAs are the main cerebral small arteries that supply blood to the BG. The atrophies of the BG and other subcortical nuclei have been reported in various neurodegenerative diseases (19, 20). The imaging data, therefore, enlightened that the atrophy of subcortical nuclei may be caused by insufficient blood supply of intracranial perforators. Further studies on the distribution of LSAs in the BG will give a general understanding of the relationship between subcortical nuclei and their supplying vessels, which helps to interpret the imaging findings and facilitate the evaluation of patients with subcortical infarcts. LSAs and other cerebral small vessels imaged by 7T TOF-MRA will add value to the studies focusing on the aging of subcortical nuclei.

In this study, all the participants received a clinical diagnosis or consultation within one month before the experiment. Subjects with hypertension, hyperlipidemia, or hyperglycemia were excluded from the recruitment. Based on the screening criterion, the decreasing trends in the number and visible length of LSA are believed to be mainly caused by normal aging. However, the data on cerebrovascular risk factors were not recorded, including the blood pressure, the concentration of blood lipids and blood glucose, body mass index, smoke history, and alcohol consumption. The relationship between the morphology of cerebral small vessels and cerebrovascular risk factors should be analyzed when quantitative data are available in future studies.

The main limitation of our study is that not all the LSAs can be visualized by 7T TOF-MRA. As a non-invasive angiographic technique that relies on the in-flow blood spins as endogenous contrast agents, only LSAs with high flow-related enhancement can be visualized by TOF-MRA, even with the voxel volume of fewer than 0.002 mm^3^ at 7T. It hinders the non-invasive observation and measurement of thinner and more distal LSAs. Moreover, only TOF-MRA was used in our current study. Future studies incorporating multimodality imaging methods such as PC-MRA and vessel wall imaging will contribute to determining whether the decreased number and length of LSAs were caused by decreased blood flow or the degenerated vasculature. In addition, only subjects over the age of 20 years were included in this study. The morphological characteristics of LSA in adolescence and early childhood are still missing. The development of LSAs and their effect on BG remains unknown, which can be meaningful in studying the development of the human brain.

## Conclusion

Overall, the morphological characteristics of LSAs are reported for the first time in a large population. The measurements of LSAs are correlated with the volume of BG nuclei. Considering the scarcity of non-invasive imaging data of cerebral small vessels, the morphological measurements of LSAs are valuable in understanding the degeneration of cerebral small vessels during aging.

## Data availability statement

The original contributions presented in the study are included in the article/supplementary material, further inquiries can be directed to the corresponding author/s.

## Ethics statement

The studies involving human participants were reviewed and approved by the Institute of Biophysics. The patients/participants provided their written informed consent to participate in this study.

## Author contributions

NW, JJ, YZ, and ZZ contributed to the conception and design of the study, analysis and interpretation of the data, and drafting of the manuscript. YZ and ZZ contributed to the funding acquisition and supervision. All authors contributed to the article and approved the submitted version.

## Funding

This work was supported by grants from the National Natural Science Foundation of China (82001804, 32000792, and 81961128030), the Youth Innovation Promotion Association CAS (2022093), the Natural Science Foundation of Beijing Municipality (7191003), the Ministry of Science and Technology of China grant (2020AAA0105601), and the Strategic Priority Research Program of the Chinese Academy of Science (XDB32010300).

## Conflict of interest

The authors declare that the research was conducted in the absence of any commercial or financial relationships that could be construed as a potential conflict of interest.

The handling editor declared a shared parent affiliation with the authors NW and JJ at the time of review.

## Publisher's note

All claims expressed in this article are solely those of the authors and do not necessarily represent those of their affiliated organizations, or those of the publisher, the editors and the reviewers. Any product that may be evaluated in this article, or claim that may be made by its manufacturer, is not guaranteed or endorsed by the publisher.
